# StarGazer: A Hybrid Intelligence Platform for Drug Target Prioritization and Digital Drug Repositioning Using Streamlit

**DOI:** 10.3389/fgene.2022.868015

**Published:** 2022-05-31

**Authors:** Chiyun Lee, Junxia Lin, Andrzej Prokop, Vancheswaran Gopalakrishnan, Richard N. Hanna, Eliseo Papa, Adrian Freeman, Saleha Patel, Wen Yu, Monika Huhn, Abdul-Saboor Sheikh, Keith Tan, Bret R. Sellman, Taylor Cohen, Jonathan Mangion, Faisal M. Khan, Yuriy Gusev, Khader Shameer

**Affiliations:** ^1^ Data Science and Artificial Intelligence, BioPharmaceuticals R&D, AstraZeneca, Cambridge, United Kingdom; ^2^ Georgetown University, Washington, DC, United States; ^3^ Biometrics, Oncology R&D, AstraZeneca, Warsaw, Poland; ^4^ Discovery Microbiome, BioPharmaceuticals R&D, AstraZeneca, Gaithersburg, MD, United States; ^5^ Early Respiratory and Immunology, BioPharmaceuticals R&D, AstraZeneca, Gaithersburg, MD, United States; ^6^ Research Data and Analytics, R&D IT, AstraZeneca, Cambridge, United Kingdom; ^7^ Discovery Sciences, BioPharmaceuticals R&D, AstraZeneca, Cambridge, United Kingdom; ^8^ Data Science and Artificial Intelligence, BioPharmaceuticals R&D, AstraZeneca, Gaithersburg, MD, United States; ^9^ Biometrics and Information Sciences, BioPharmaceuticals R&D, AstraZeneca, Mölndal, Sweden; ^10^ Neuroscience, BioPharmaceuticals R&D, AstraZeneca, Cambridge, United Kingdom

**Keywords:** multi-omics, target prioritization, drug discovery, repositioning, data integration, streamlit, stargazer, hybrid intelligence

## Abstract

Target prioritization is essential for drug discovery and repositioning. Applying computational methods to analyze and process multi-omics data to find new drug targets is a practical approach for achieving this. Despite an increasing number of methods for generating datasets such as genomics, phenomics, and proteomics, attempts to integrate and mine such datasets remain limited in scope. Developing hybrid intelligence solutions that combine human intelligence in the scientific domain and disease biology with the ability to mine multiple databases simultaneously may help augment drug target discovery and identify novel drug-indication associations. We believe that integrating different data sources using a singular numerical scoring system in a hybrid intelligent framework could help to bridge these different omics layers and facilitate rapid drug target prioritization for studies in drug discovery, development or repositioning. Herein, we describe our prototype of the StarGazer pipeline which combines multi-source, multi-omics data with a novel target prioritization scoring system in an interactive Python-based Streamlit dashboard. StarGazer displays target prioritization scores for genes associated with 1844 phenotypic traits, and is available via https://github.com/AstraZeneca/StarGazer.

## Introduction

Drug repositioning has been rapidly gaining attention in the drug discovery domain during the past decade (Xue et al., 2018). Drug repositioning/repurposing describes the act of identifying alternative uses for a drug beyond the scope of its original indication, regardless of whether it has been FDA-approved or has failed in clinical trials (Pushpakom et al., 2019). The reasons for investing into drug repositioning are very numerous indeed.

Traditionally, a standard drug development cycle is estimated to take around 10 years and requires billions of dollars of investment, notwithstanding the still disappointingly high failure rate at clinical trials (Li et al., 2016). In light of these problems, drug repositioning holds potential to drastically reduce the time and money needed to bring a drug to the market: it has been estimated to reduce the time by half and cut costs by 5-fold when compared to developing a new drug from scratch (Shameer et al., 2015). These factors alone highlight the appealing opportunity to bring medicines to patients faster, and potentially into areas of unmet therapeutic demand. Moreover, it allows for the existing arsenal of approved drugs to be more broadly utilized, and for the opportunity to salvage some costs involved in the development of drugs that failed in clinical trials. Finally, the sheer variety in successful and promising repositioning strategies to date speaks to the potential for unearthing profound biological links between different diseases, driving paradigm shifts in our approach to modern medicine (Lee and Bhakta, 2021).

Drug target prioritization is an essential step for repositioning as it aims to highlight the potential drug targets for a particular disease. Applying computational methods to analyze and process multi-omics data is an effective approach for achieving this (Ashburn and Thor, 2004; Glicksberg et al., 2014; Shameer et al., 2018a; Pushpakom et al., 2019; Guo et al., 2021; Rapicavoli et al., 2022). Whilst there is now a vast wealth of biochemical and biomedical data in the current era of high-throughput omics technology, our ability to integrate and interpret these data has lagged behind and is presenting a great challenge in disease biology (Shameer et al., 2015). While machine learning approaches are generally used to develop tools to integrate, analyze and interpret multi-omics data, it remains a challenge that mere automation of predicting biological insights might overrepresent hypotheses that cannot be validated using function test experiments (Hodos et al., 2016; Peters et al., 2017). In such a scenario, we recommend the application of a hybrid intelligence platform that enables visual intelligence, quick search, contextual interpretations with quantitative approaches as a way to address this problem. Hybrid intelligence systems have been developed to address challenging problems in biomedicine, including remote patient diagnosis (Abu-Doleh et al., 2012; Li et al., 2014a; Akata et al., 2020; Guo et al., 2021; Weissler et al., 2021). However, such approaches are not readily available to address challenges in data integration and mining associated with drug target prioritization and drug repositioning.

Data from genome-wide association studies (GWAS) and phenome-wide association studies (PheWAS) have been used for drug target prioritization (Ferrero and Agarwal, 2018). Whilst GWAS aim to identify associations between genetic variants with a single phenotype, PheWAS interrogate numerous phenotypic traits at once (Denny et al., 2010). As of 06 October 2021, the EMBL-EBI GWAS catalog collates associations from 5,370 studies that, in total, identified more than 290,000 associations. The utility of this GWAS dataset can be further amplified by narrowing down the genes of interest to only those with known drug indications (Sanseau et al., 2012). Importantly, a three-step strategy for drug repositioning using PheWAS data has already been proposed (Rastegar-Mojarad et al., 2015): (Xue et al., 2018)—identify all genes with known associations with the phenotypic trait of interest using PheWAS data; (Pushpakom et al., 2019);—identify all drugs with associations with the previously identified genes using data from DrugBank; and (Li et al., 2016)—return all the drugs identified in the previous step as candidates for repositioning for the original phenotypic trait of interest. Others have gone further by incorporating a combination of data from GWASs (Khosravi et al., 2019), expression profile analysis (Lau and So, 2020), functional annotation, biological network analysis, and gene-set association (Reay and Cairns, 2021).

Taken together, these data highlight the potential of using GWAS and PheWAS data for drug target prioritization. However, the field is still young, and integrating disparate data sources remains relatively limited in scope (Gallo et al., 2021). We hypothesize that integrating multimodal data sources using a singular numerical scoring system could accelerate the discovery and prioritization of drug targets. In light of this, we present our interactive dashboard, StarGazer, which aims to address these challenges by integrating three different datatypes (i.e., disease-target association, target druggability, and target protein-protein interaction) into a novel scoring system, utilizing real-time API calls and Python-based Streamlit technology. While these types of datasets have been used for numerous repositioning studies separately (Liu et al., 2014; Khaladkar et al., 2017; Hermawan et al., 2020; Wijetunga et al., 2020; Adikusuma et al., 2021; Attique et al., 2021; Ghoussaini et al., 2021; Portelli et al., 2021; Tan et al., 2021; Varghese and Majumdar, 2022; Zhao et al., 2022), StarGazer represents the first ever integration of the PheWAS catalog, Open Targets, STRING and Pharos, all of which are well-curated, well-studied, open access databases. Furthermore, computational repositioning studies focus largely on singular diseases, phenotypes or drugs, but StarGazer is equipped for flexible investigation into any of the 1,844 phenotypes and traits within the dashboard. Much of the data is up-to-date with the latest science, as it is loaded in real-time before it is analyzed in real time. StarGazer’s drug target prioritization mode allows for rapid identification of potential drug targets for a disease of interest, also providing immediate analysis of various aspects surrounding drug development, such as druggability and the nature of the target-disease association. In addition to this target prioritization feature, we anticipate that StarGazer’s ability to display all phenotypes associated with genes or gene variants of interest in an easily digestible manner to be of great value to exploratory or analytical workflows. Furthermore, StarGazer’s other features include the support of initial discoveries by interrogating the precise contribution of evidence from each data source.

## Data

Disease-target data are acquired from the PheWAS catalog (https://phewascatalog.org/phewas) and OpenTargets (https://genetics.opentargets.org/). The latest PheWAS catalog was created in 2013 by generating odds ratios of association between 3,144 SNPs identified in GWASs and 1,358 phenotypes derived from the electronic medical records of 13,835 individuals of European ancestry, and the data is loaded locally (Denny et al., 2013). The list of phenotypic variants from the PheWAS catalog as well as from the GWASs within the PheWAS catalog were aggregated and filtered to remove duplicates, producing a list of 1844 phenotypic traits which StarGazer uses for subsequent analysis. OpenTargets version 22.02 is the latest version at the time of writing, and provides 7,980,448 target-disease association scores extracted from 21 public databases containing diverse forms of evidence, from genetic and drug associations to text mining and animal model data amongst others (Ochoa et al., 2021). Data from OpenTargets is acquired in real-time via API calls.

Target druggability data are acquired in real-time via API calls through Pharos (https://pharos.nih.gov/) to access the Target Central Resource Database (TCRD) (Sheils et al., 2021). The TCRD categorizes 20,412 targets, at the time of writing, into four groups of increasing druggability evidence: Tdark, Tbio, Tchem, and Tclin. A variety of evidence is integrated for classification, such as data from ChEMBL (Mendez et al., 2019), Guide to Pharmacology (Armstrong et al., 2019), DrugCentral (Avram et al., 2021), and antibodypedia (Kiermer, 2008), amongst many more, as well as gene ontology and text-mining analysis. Tclin genes are already targets of approved drugs, whilst Tchem genes have drugs with evidence of sufficient activity against the gene. Tbio genes have weak evidence for druggability, and Tdark genes have an unknown level of druggability.

Protein-protein interaction data are acquired in real-time via API calls from the STRING database (https://string-db.org/). STRING version 11.5 contains data of 20,052,394,042 protein-protein interactions from 14,094 organisms, of which only human genes and orthologous genes were used in StarGazer (Szklarczyk et al., 2021), which were analyzed using the Python package, pyvis. Gene ontology enrichment analysis is also performed by STRING.

## Methods

**FIGURE 1:**
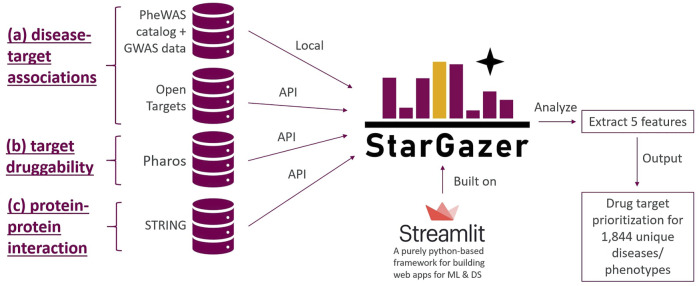
The StarGazer drug target prioritization framework considers the following five features for each of the 1844 diseases in StarGazer’s disease list (Xue et al., 2018):—the odds ratios of association between targets and phenotypic variants of interest from GWAS and PheWAS data (Pushpakom et al., 2019);—the target-disease association scores from Open Targets (Li et al., 2016);—the druggability data of genes of interest from Pharos (Shameer et al., 2015);—the degree of nodes in protein-protein interaction networks of genes of interest from STRING; and (Lee and Bhakta, 2021)—the presence of the gene variant of interest in both PheWAS and GWAS datasets. All data, except the PheWAS and GWAS data, are loaded in real-time by API calls and therefore present the latest evidence for drug repositioning strategies. The above five features are then integrated to provide a singular numerical StarGazer score which quantifies the drug repositioning potential of a gene. StarGazer is built on the Python-based Streamlit platform, which is largely used for building sleek and modern web applications for machine-learning and data science.

StarGazer was built using Streamlit (https://streamlit.io/), a relatively new Python-based tool for developing web applications for machine learning and data science. It enables data scientists to build web applications purely from Python scripts quickly and seamlessly. The Streamlit dashboard allows for local files to be loaded, as well as data to be requested from databases via real-time API calls. The StarGazer drug target prioritization framework considers the following five features for each disease (Figure 1): (Xue et al., 2018)—the odds ratios of association between targets and phenotypic variants of interest from GWAS and PheWAS data; (Pushpakom et al., 2019);—the target-disease association scores from Open Targets; (Li et al., 2016);—the druggability data of genes of interest from Pharos; (Shameer et al., 2015);—the degree of nodes in protein-protein interaction networks of genes of interest from STRING; and (Lee and Bhakta, 2021)—the presence of the gene variant of interest in both PheWAS and GWAS datasets. Each gene was analyzed with respect to each of these five features, and five scores were computed corresponding to each of the above features. These five scores were then normalized to ensure equal maximum contribution, before summing the five normalized scores to obtain an overall score (i.e., the StarGazer score) which has a maximum score of 1. The targets were then ranked in descending order to facilitate target prioritization.

### Processing of Disease-Target Data

Analysis of the PheWAS and GWAS odds ratios involved identifying risk associations where the odds ratio ≥1 (i.e., more associated with the occurrence of the phenotype), and protective associations where the odds ratio <1 (i.e., more associated with the non-occurrence of the disease). In the risk allele-based target prioritization, odds ratios were taken as they were. However, in protective-allele-based target prioritization, odds ratios were subtracted by 1, as the lower ratio implies higher magnitude of association. An average value was taken for odds ratios from multiple studies of the same gene, before normalizing to generate the feature score. Another feature score was generated by determining if the gene target was present in both the PheWAS and GWAS datasets, assigning a score of 1 for the PheWAS-GWAS intersection score, which is otherwise 0. Finally, the target-disease association feature scores from OpenTargets were values between 0 and 1, calculated in a similar manner as the PheWAS catalogue analysis.

### Processing of Target Druggability Data

For analysis of the druggability data from Pharos, the number of distinct druggability levels that a target has was counted, with the exception of Tdark, e.g., a target with Tbio, Tclin, and Tdark labels is scored 2 (1 + 1 + 0). These scores were then normalized against the highest druggability feature score of each gene.

### Processing of Protein-Protein Interaction Data

The degree of the node in the protein-protein interaction networks from STRING is the number of proteins directly connected to the target node via functional associations, which include experimentally confirmed interactions, predicted interactions and text mining data. Node degrees were computed for each gene in a network and calculated as a ratio of the highest node degree in that network, as a gene with higher interactivity within a STRING network is more likely to be biologically underpinning the molecular pathway that contributes to a phenotype. The calculation of node degrees scores this way also reduces effects of false positive interactions.

## Results

**FIGURE 2:**
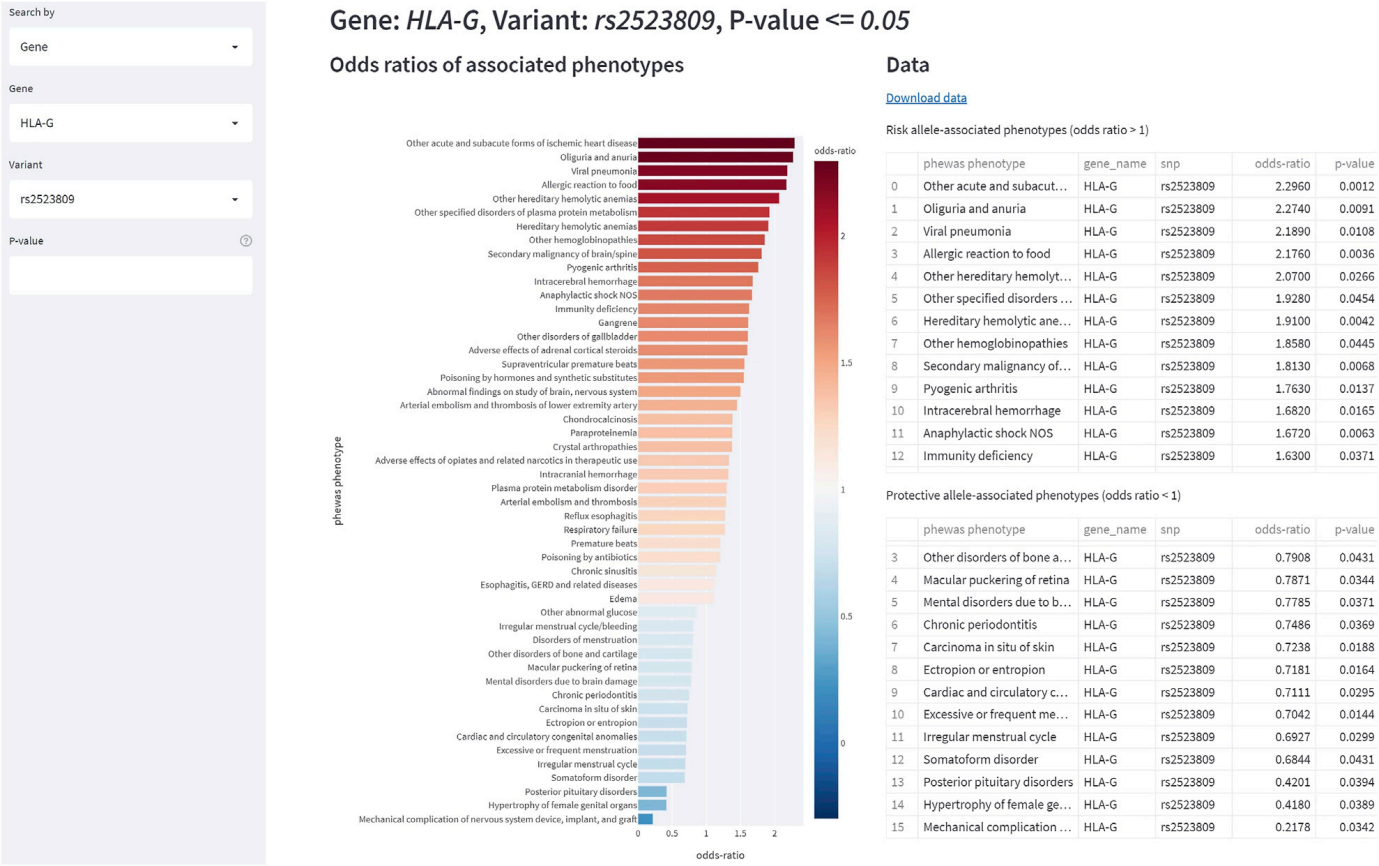
The StarGazer interface after searching “*HLA-G*” in Gene mode. At *p* = 0.05, the first allele returned is rs11206510. The color-coded bar chart shows the odds ratio of association of the allele with each phenotype. The table on the right is the same data tabulated which can be downloaded as a csv file. The StarGazer Variant mode is similar in appearance.

**FIGURE 3:**
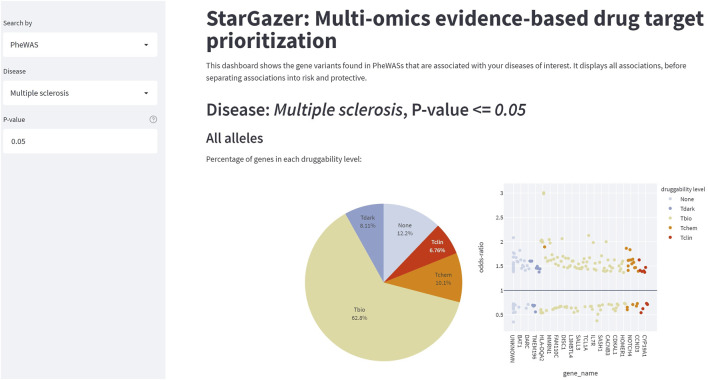
The StarGazer interface after searching “Multiple sclerosis” in PheWAS mode. At *p* = 0.05, 7.37% of genes with associations with multiple sclerosis were categorized as Tclin, i.e., already targets of FDA-approved drugs. The distribution of genes in each druggability level is shown by pie chart and scatter plot, the latter of which also showing the odds ratios of each allele of each gene. Some gene names are not shown. This data is re-analyzed to show only risk alleles, or only protective alleles. Tabulated data can be visualized and downloaded. The StarGazer modes, GWAS and GWAS-PheWAS Union, are similar in appearance.

**FIGURE 4:**
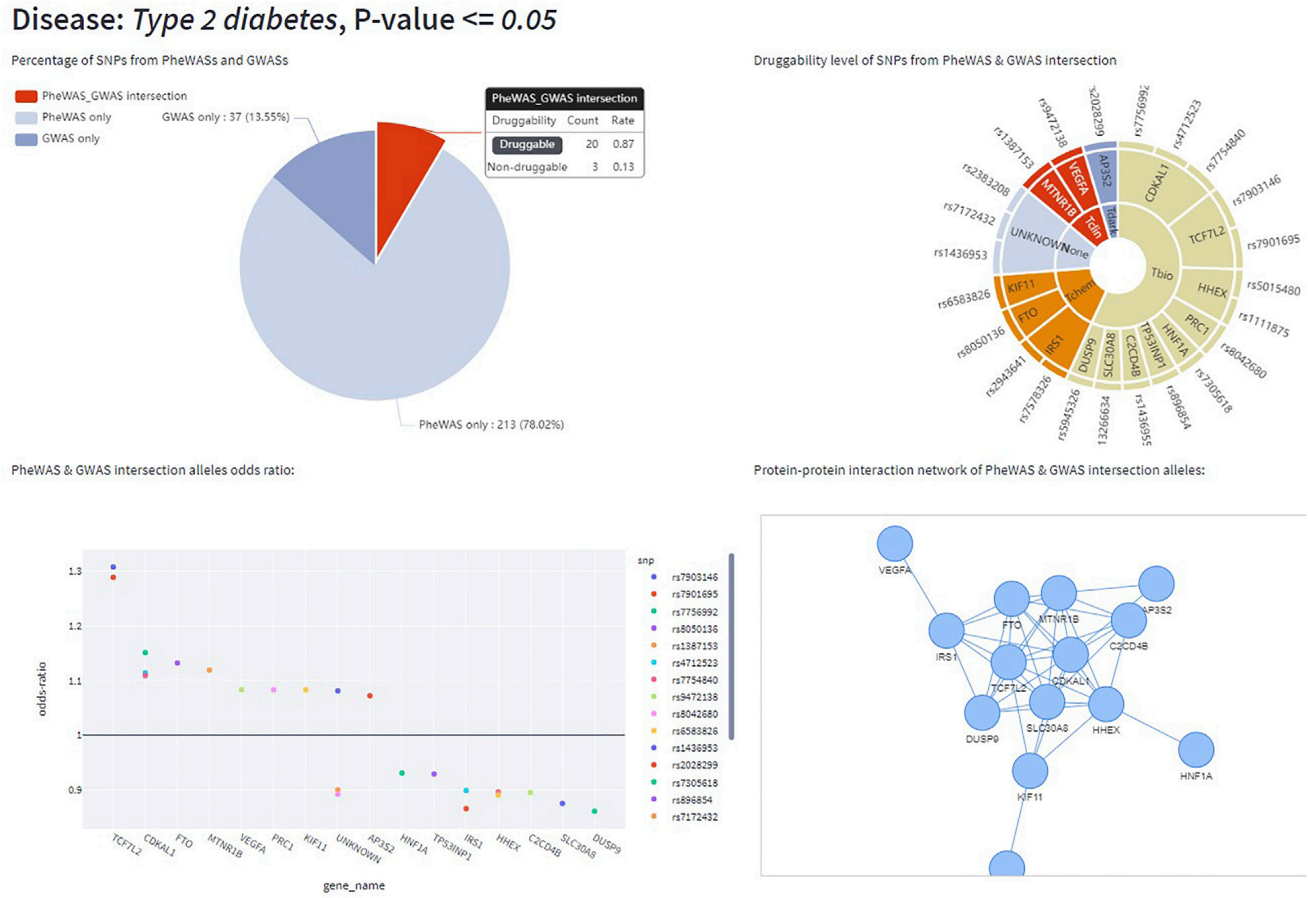
The StarGazer interface after searching “Type 2 diabetes” in GWAS-PheWAS Intersection mode. At *p* = 0.05, 23 SNPs were identified to have associations in both PheWASs and GWASs. Top left: pie chart displaying the proportion of SNPs that were identified in either PheWAS or GWAS datasets, or in both datasets. Top right: pie chart displaying druggability information of the genes of these SNPs. Tclin in red implies genes already have drugs targeting them available on the market, whilst Tchem, Tbio, Tdark, and None, indicate progressively decreasing levels of druggability. Bottom left: scatter plot highlighting individually reported odds-ratios of associations of SNPs from various GWASs. Bottom right: a protein-protein interaction network constructed from the genes of alleles detected in both GWASs and the PheWAS catalog. The gene ontology enrichment analysis feature is not shown in the figure.

**FIGURE 5:**
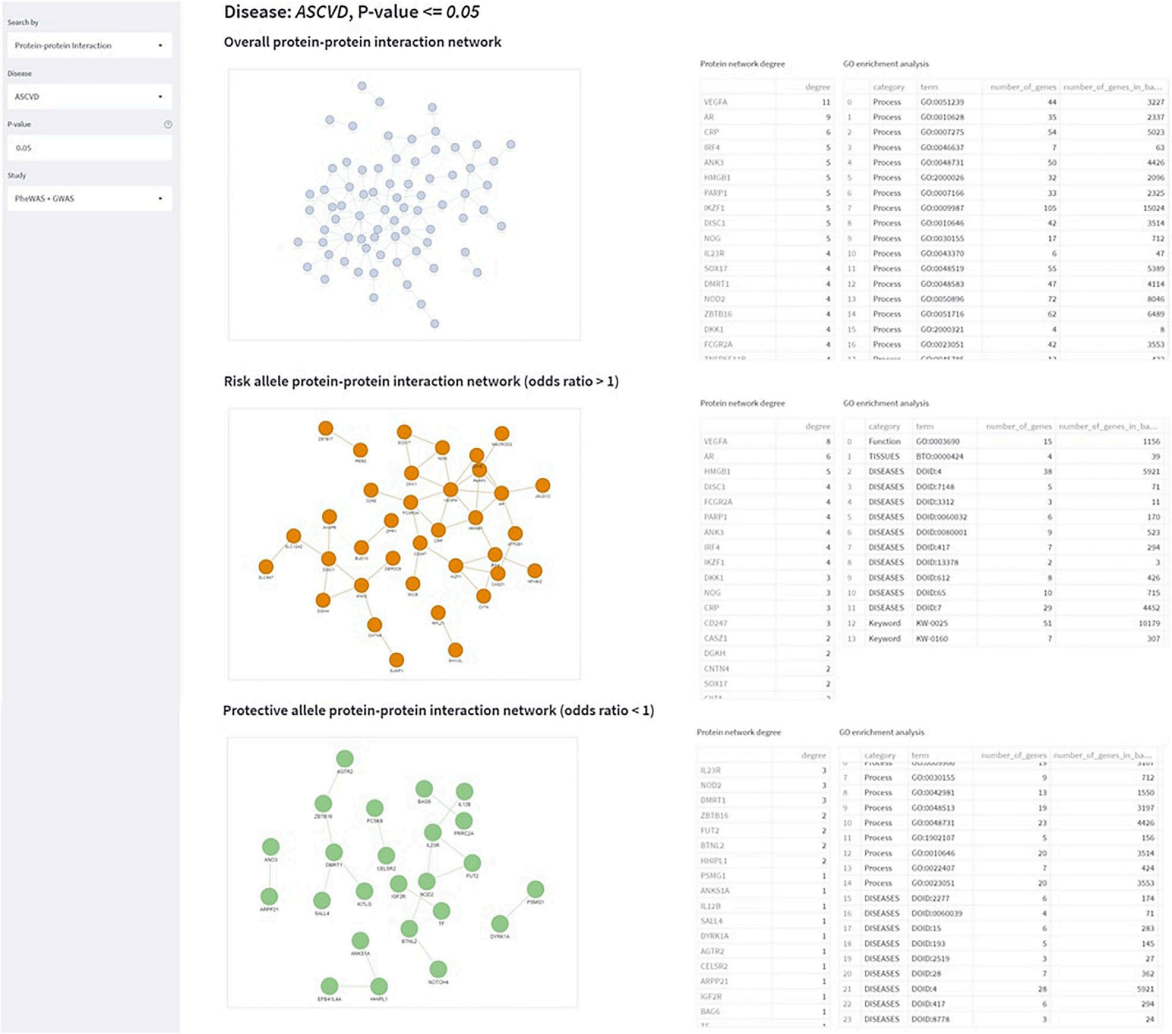
The StarGazer interface after searching “ASCVD” in Protein-protein interaction mode. Protein-protein interaction networks are shown of all alleles, risk alleles, and protective alleles. The node degree of the genes of these alleles are computed, and gene ontology enrichment analysis is performed on the right.

**FIGURE 6:**
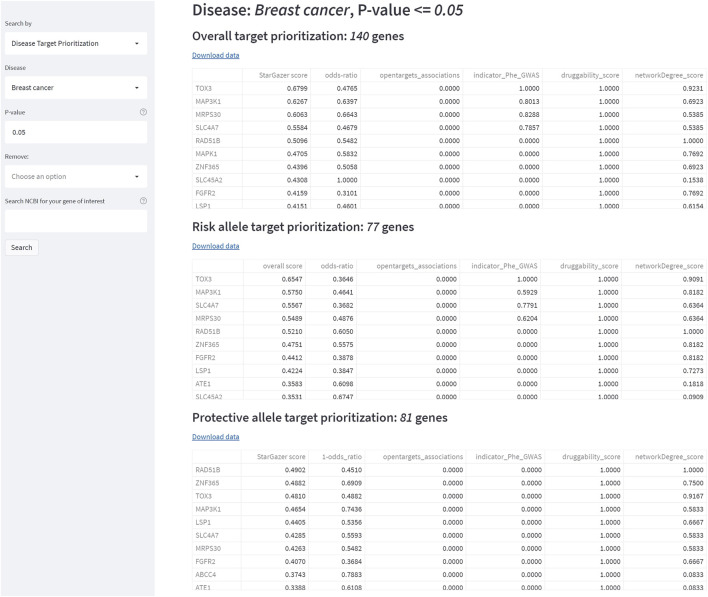
The StarGazer interface after searching “Breast cancer” in Disease Target Prioritization mode. At *p* = 0.05, 140 genes are returned to have association with breast cancer. Genes are ranked in StarGazer score, which describes how suitable a gene is for drug repositioning. The subsequent five columns are the individual scores of the five features extracted from all of the data that contribute to the StarGazer score. Data are separated into all alleles, risk alleles, then protective alleles, and can be downloaded as csv files.

The StarGazer dashboard (https://github.com/AstraZeneca/StarGazer) offers eight modes of data exploration for drug target prioritization using the data analyzed as described in Methods. The modern yet simple interface allows for rapid navigation without the need for specialist training or programming experience. StarGazer allows users to search by genes or gene variants which displays all associated phenotypic variants ranked by odds ratio graphically, as well as in tabular format (Figure 2). Red bars indicate an odds ratio of greater than 1 (i.e., risk association), whilst blue bars indicate less than 1 (i.e., protective association). Users can also search by the PheWAS, GWAS, and GWAS-PheWAS Union modes of exploration, which returns odds ratios of all variants of genes associated with the phenotype of interest from the respective datasets, as well as their corresponding druggability levels (Figure 3). When searching in the GWAS-PheWAS Intersection mode, only variants with associations identified in both GWAS and PheWAS datasets are shown (Figure 4). For these variants, the dashboard also provides association odds ratios, druggability data, protein-protein interaction networks and gene ontology enrichment analysis for the disease of interest (Figure 5). Finally, when users search by disease target prioritization, the overall StarGazer score is shown for each gene with association with the disease of interest (Figure 6). Contextual information on any of these genes can be found immediately using the build-in NCBI search tool. For each of these exploration modes, users can also modify the *p*-value to only display associations of desired statistical significance assigned by the origin data source.

### Use Case: StarGazer for Understanding Complex Diseases

In the following case study, we posed as someone who was simply curious about the possible mechanistic causes of insomnia, and consequently adopted a more exploratory workflow. As insomnia is a complex and relatively understudied disorder, we set the *p*-value to a less stringent 0.05 to prevent issues in, for example, study sample size or sensitivity from masking any potentially true associations. This returned a list of 106 genes with associations with insomnia, 62 of which had at least one risk-associated allele, and 46 had at least one protection-associated allele (Table 1). After searching on NCBI, there were three genes found to have significant relevance to insomnia. *DISC1* encodes a scaffold protein which is involved in brain development, and its mutations have been implicated in schizophrenia and other psychiatric disorders (Dahoun et al., 2017); *MAOA* encodes a mitochondrial oxidative deaminase targeting amines such as dopamine, norepinephrine, and serotonin, and mutations in the gene can result in Brunner syndrome, a psychiatric and sleep disorder (Brunner et al., 2007); *MEIS1* is a HOX gene thought to have a pleiotropic effect on chronic insomnia disorder, and have possible association with restless leg syndrome (Sarayloo et al., 2019). We also found genes with a variety of functions and unclear links with insomnia. Tumor suppressor genes, *CMTM7* (Li et al., 2014b), *NKAPL* (Okuda et al., 2015) and *ATM* (encoding ATM checkpoint kinase) (Shiloh and Ziv, 2013) may allude to aberrant DNA damage responses contributing to insomnia, and indeed, there are several reports of links between DNA damage and sleep in the literature (Carroll et al., 2016; Zada et al., 2021). HLA isoforms indicate a potential immunity-related cause of insomnia (Choo, 2007). *In vitro* mutants in vesicular trafficking protein, dynamin-1, have impaired ability to recycle neurotransmitter at synapses (Chung et al., 2010), providing a more obvious potential link with insomnia. Finally, genes with noticeably pleiotropic effect were also found to have a high StarGazer score. One such example is estrogen receptor (*ESR1*), important for gestation in women but is in addition expressed in many non-reproductive tissues in both sexes, as it has roles more broadly in growth and metabolism (Barros and Gustafsson, 2011). Not only is estrogen receptor linked with breast cancer but also with osteoporosis (Gennari et al., 2007), and thus makes for a peculiar hit on the StarGazer analysis. Although additional investigations are required to ascertain the link between these genes and phenotypes, it is exciting to hypothesize about the underlying molecular mechanisms. This is especially the case for insomnia, a disorder of sleep which is a biological process we still have a relatively poor understanding of.

**TABLE 1:** Top 30 hits from Disease Target Prioritization mode analysis of “Insomnia” using StarGazer.

Gene Name	StarGazer Score	Odds-Ratio	OpenTargets Associations	Indicator Phe/GWAS	Druggability Score	Network Degree Score
HLA-DRB1	0.456	0.725	0.000	0.000	1.000	0.556
ESR1	0.433	0.655	0.009	0.000	0.500	1.000
GRIN2B	0.407	0.756	0.000	0.000	0.500	0.778
MEIS1	0.395	0.251	1.000	0.000	0.500	0.222
MAOA	0.344	0.888	0.000	0.000	0.500	0.333
DNM1	0.337	0.630	0.000	0.000	0.500	0.556
HLA-DQB1	0.320	0.653	0.000	0.000	0.500	0.444
BMP4	0.307	0.591	0.000	0.000	0.500	0.444
ATM	0.293	0.188	0.000	0.000	0.500	0.778
CMTM7	0.288	0.941	0.000	0.000	0.500	0.000
NKAPL	0.288	0.938	0.000	0.000	0.500	0.000
GRIA1	0.286	0.263	0.000	0.000	0.500	0.667
TOMM40	0.280	0.677	0.000	0.000	0.500	0.222
NR5A2	0.278	0.668	0.000	0.000	0.500	0.222
HDAC9	0.276	0.768	0.000	0.000	0.500	0.111
MS4A6A	0.271	0.631	0.000	0.000	0.500	0.222
DISC1	0.267	0.166	0.000	0.000	0.500	0.667
ST6GAL1	0.265	0.716	0.000	0.000	0.500	0.111
SLC22A3	0.264	0.600	0.000	0.000	0.500	0.222
EFNA5	0.264	0.600	0.000	0.000	0.500	0.222
NRGN	0.263	0.706	0.000	0.000	0.500	0.111
DRD2	0.263	0.000	0.150	0.000	0.500	0.667
RNASET2	0.263	0.591	0.000	0.000	0.500	0.222
FGFR2	0.263	0.257	0.000	0.000	0.500	0.556
UBE2L3	0.262	0.701	0.000	0.000	0.500	0.111
YDJC	0.260	0.690	0.000	0.000	0.500	0.111
CDC42BPB	0.260	0.690	0.000	0.000	0.500	0.111
LAMP3	0.258	0.791	0.000	0.000	0.500	0.000
ARG1	0.254	0.660	0.000	0.000	0.500	0.111
CCND3	0.251	0.643	0.000	0.000	0.500	0.111

## Discussion

StarGazer is a novel application built for rapid investigation of drug repositioning strategies. It combines multi-source, multi-omics data with a novel target prioritization scoring system in an interactive Python-based Streamlit dashboard. StarGazer analyzes and integrates disease-target associations, druggability data, and protein-protein interaction data before extracting five features from the data to create an overall StarGazer score for every potential target associated with StarGazer’s curated list of 1844 phenotypic variants.

StarGazer is adapted to facilitate exploration of the human biology landscape from a birds-eye view, allowing rapid digestion of information from PheWASs/GWASs, which otherwise contains many tens of thousands of complex multivariate datapoints. Streamlit, as a user interface package adapted for complex data visualization and user interactivity, was considered to be a well-suited technology for such a task. Indeed, the importance of the flexibility in visualization methods, and live data retrieval and analysis is becoming increasingly clear, with their applications ever-expanding (Badgeley et al., 2016; Moosavinasab et al., 2016).

We demonstrate the utility in integrating several omics datasets and returning easy-to-interpret analysis metrics in an interactive dashboard. One can easily imagine the power of such a strategy as we incorporate state-of-the-art, machine learning-based, multi-omics integration techniques, as well as a wider variety of high quality data. In an era where the speed at which we can generate data is accelerating at a higher rate than we can analyze it, we anticipate that integrative scores and visualization tools will grow increasingly essential in biology, and that we must begin to break away from the more rigid, single-use analysis framework that forms the modern paradigm for analyzing not just GWAS and PheWAS data (Diogo et al., 2018; Ferrero and Agarwal, 2018; Robinson et al., 2018; Lau and So, 2020), but multi-omics data in general (Subramanian et al., 2020).

StarGazer has been built with the goal of pushing multi-omics integration towards upward scalability by providing users with immediate access to contextual information on genes of potential interest by automatically performing several steps of follow-up analysis on all genes - this saves a considerable amount of time from performing speculative follow-up analysis. These follow-up analysis steps are completed in bulk through the processing of the single-omic layers, which removes the need for users to analyze every gene separately for various properties and then later compare the results to make sense of the evidence. Not only does integrating single-omic layers increase the speed of exploratory data analysis, but it also provides additional value from combining multiple pieces of evidence as opposed to focusing on individual single high-confidence pieces of information, especially when the different types of data are likely to have an intimate biological relationship, e.g., combining a gene’s DNA, RNA and protein information together is likely to be more valuable than analyzing them independently as they are functionally coupled. This approach may be our best strategy for uncovering complex and profound relationships and hence, the phrase “the whole is greater than the sum of its parts” holds particularly true in the context of multi-omics data analysis. A more integrated strategy may also be more useful in helping us understand the genetic basis of complex diseases driven by genes and gene variants with pleiotropic functions or effects. Applying the latest ideas on pleiotropy in biological systems to future work may allow us to obtain a more complete understanding of genome-phenome relationships and thus drive novel discoveries previously inaccessible in the biomedical field (Shameer et al., 2021).

### Limitations

This should, of course, highlight to the reader the current co-dependence between broader exploratory analytical approaches, such as StarGazer, with those that possess stronger statistical power, aimed at target confirmation at the cost of breadth and fewer omics layers, and of course, experimental confirmation. Moving forwards, we should hope that the field develops more sophisticated strategies for these types of analysis. All in all, we anticipate StarGazer to be potentially useful in providing insights into many types of biological pathways, as long as the molecular perturbations that are linked with disease lie close to the genetic level. Whilst it is easy to imagine StarGazer’s utility for studying diseases caused by variants of proteins or nucleic acids due to their more direct connection to genome-level information, studying metabolic disorders of carbohydrates and lipids would be possible but more difficult.

We wish to highlight that, although the barrier to entry for multi-omics data analysis is low, there seems yet a limitless space for improvement in the field at the time of writing. In the future, we aim to incorporate gene ontology terms enrichment analysis, gene semantic similarity, and gene expression data into our target prioritization framework, and improve on the implementation of protein-protein interaction networks (Shameer et al., 2016; Peters et al., 2017). Whilst the current version of StarGazer extracts several features for target-disease associations, the assessment of target druggability uses only one dataset to generate one feature. Although the knowledge-based classification of the genome that Pharos provides is very high quality data, it is less indicative of future potential developments as it reflects only the current status of the druggability landscape of human biology. Therefore, more predictive datasets, such as computational docking predictions using structural data from molecular techniques or even AI-based computational prediction, may provide more robust insight into the future (Baek et al., 2021; Jumper et al., 2021).

StarGazer’s use of API calls allows for the majority of its data to be updated automatically with the latest relevant studies, aside from the PheWAS catalog which was performed in 2013—it would be invaluable if a similar study was repeated to include the GWAS data which was generated during the decade that has elapsed since the original effort. Furthermore, a variety of machine learning strategies have been applied to multi-omics data analysis and show great promise in assisting precision medicine and repositioning (Shameer et al., 2018b; Nicora et al., 2020; Reel et al., 2021), and is therefore an area we are interested in developing for StarGazer. Another avenue for future development is to improve on the standardization of clinical terms between the different datasets, which is a problem not unique to StarGazer but found ubiquitously in healthcare-related work (Wears, 2015; Beck et al., 2019). This problem manifested itself as data from OpenTargets being underrepresented in the overall StarGazer score. We hypothesize that using a combination of standardized codes for clinical terms, such as ICD-9/-10 (https://www.cdc.gov/nchs/icd/icd9.htm, https://www.cdc.gov/nchs/icd/icd10.htm), and EFO (https://www.ebi.ac.uk/efo/faq.html), would help with this problem, as well as further curate our list of 1844 phenotypic variants. Currently, the code for installation can be found on GitHub (https://github.com/AstraZeneca/StarGazer).

## Conclusion

We have created StarGazer (https://github.com/AstraZeneca/StarGazer), an interactive dashboard that facilitates rapid investigation of potential novel drug targets and repositioning strategies. It integrates three different types of data (disease-target data, target druggability data, and protein-protein interaction data) from four different knowledgebases (the PheWAS catalog, OpenTargets, Pharos, and STRING) to extract five features that are then processed to return a singular normalized “StarGazer” score. All genes with associations with any of the 1844 phenotypic variants in the StarGazer disease list are then ranked in suitability for drug repositioning strategies for the disease of interest.

We demonstrate the utility in integrating several omics datasets to return easy-to-interpret analysis metrics in an interactive dashboard. One can easily imagine the power of such a strategy as we incorporate machine learning techniques as well as a wider variety of high quality data. It is anticipated that such integrative analysis strategies will become commonplace as biomedical data science grows to explore more multi-disciplinary and multi-omic datasets. Integrative scores and visualization tools for high dimensional data will become essential as we navigate science in this era where we are generating data at a such an enormous pace, thus we have positioned StarGazer to push multi-omics integration towards upward scalability.

## Data Availability

The datasets presented in this study can be found in online repositories. The names of the repository/repositories and accession number(s) can be found below: https://github.com/AstraZeneca/StarGazer.
